# Correlated Skin Surface and Tumor Motion Modeling for Treatment Planning in Robotic Radiosurgery

**DOI:** 10.3389/fnbot.2020.582385

**Published:** 2020-11-12

**Authors:** Shumei Yu, Pengcheng Hou, Rongchuan Sun, Shaolong Kuang, Fengfeng Zhang, Mingchuan Zhou, Jing Guo, Lining Sun

**Affiliations:** ^1^School of Mechanical and Electrical Engineering, Soochow University, Suzhou, China; ^2^Computer Aided Medical Procedures, Technical University of Munich, Munich, Germany; ^3^School of Automation, Guangdong University of Technology, Guangzhou, China

**Keywords:** respiratory motion characterization, voxel model, correlation model, tumor tracking, surface modeling

## Abstract

In robotic radiosurgery, motion tracking is crucial for accurate treatment planning of tumor inside the thoracic or abdominal cavity. Currently, motion characterization for respiration tracking mainly focuses on markers that are placed on the surface of human chest. Nevertheless, limited markers are not capable of expressing the comprehensive motion feature of the human chest and abdomen. In this paper, we proposed a method of respiratory motion characterization based on the voxel modeling of the thoracoabdominal torso. Point cloud data from depth cameras were used to achieve three-dimensional modeling of the chest and abdomen surface during respiration, and a dimensionality reduction algorithm was proposed to extract respiratory features from the established voxel model. Finally, experimental results including the accuracy of voxel model and correlation coefficient were compared to validate the feasibility of the proposed method, which provides enhanced accuracy of target motion correlation than traditional methods that utilized external markers.

## Introduction

Robotic radiosurgery shows significant advantages in treating tumors that are not suitable to be treated by chemotherapy (Coste-Maniere et al., 2005). Since control of radioactive beam according to tumor motion tracking ensures treatment of high precision, accurate tumor respiratory motion tracking is crucial for stereotactic radiosurgical robots. Movement of tumors caused by respiration is complex to be modeled; usually movement of tumors is obtained by X-ray imaging. However, the method of locating tumors *in vivo* by frequent irradiation of X-rays causes unnecessary secondary damage to normal tissues. Therefore, correlating the skin motion with tumor motion simultaneously caused by respiration is necessary before establishing a prediction model to obtain the upcoming position of tumor.

To develop a treatment planning system considering tumor motion caused by human breath, respiratory motion characterization of the skin surface is needed first. Several methods have been developed to model the respiratory skin motion. According to surrogate variations, researchers have studied the correlation of tumor movements with motion of markers on the chest and abdomen surface, multimodal (airflow, tidal volume, acceleration, force) features, and motion measurement of the chest and abdomen surface, respectively.

Currently, placing markers on the skin surface is the mostly adopted way to study the respiration characterization and external–internal motion correlation. In the study of the characterization of respiratory motion with a single marker, the motion of one marker on the abdominal skin and tumor was fitted as a linear relationship *in vivo*, but insufficient characterization in experiments was found (Kanoulas et al., 2007). CyberKnife (Cheng and Adler, 2006) used three markers to record the motion of skin surface in respiratory tracking system. Nevertheless, if the patient performs complex breathing pattern, limited surrogates could not reflex the complicated respiration mode. The correlation models between three markers and tumor *in vivo* based on linear/quadratic fitting, artificial neural network, and fuzzy logic were compared, respectively, and the results found that the correlation coefficient of fuzzy logic algorithm is higher (Torshabi et al., 2013). The tumor tracking method proposed (Iizuka et al., 2015) is based on a pre-established 4D model, which connects internal tumor motion with external respiratory signals. Respiratory signals were collected by monitoring four infrared (IR) markers on the abdominal wall of the patient using an IR camera. The method can effectively reduce the influence of radiotherapy on normal tissues and further provides local control. However, due to insufficient characterization of respiratory signals by a limited number of IR markers, there was inevitable accumulation error of targeting in the abdominal region. The correlation factors between four surface markers and the tumor in different directions of motion were analyzed (Koch et al., 2004). Studies have shown that the association degree of skin surface markers depends on the placement of markers and the breathing pattern of patients. In the study of six markers, the centroid motion curves of all markers and the corresponding tumor motion curves were calculated; finally, tumor locations by proportional linear interpolation were obtained (Schweikard et al., 2000). Furthermore, 19 infrared LED markers on abdominal skin of swine were adopted to study the respiration feature and proved that the more markers utilized on the skin, the more respiratory movement information can be extracted (Ernst et al., 2009).

For the study of multimodal information of the chest and abdomen surface, researchers studied the linear correlation factors of tidal volume, abdominal surface displacement, and the anterior–posterior displacement of tumor during human respiration and concluded that tidal volume has higher correlation with tumor displacement (Hoisak et al., 2004). However, the correlation between tidal volume and tumor has a noticed drift with respiratory phase, and the measurement of tidal volume brings great pain to patients. In addition, multimodal information using tension bands, optical markers, acceleration sensors, airflow acceleration, and temperature sensors was collected to compare their correlation effects of inner–outer respiratory motion and found that optical marker achieves the best performance for the tumor motion tracking (Durichen et al., 2013).

For the study of correlating tumor motion with chest and abdomen surface motion, there have been some exploratory researches on the measurement of the whole thoracoabdominal surface motion. A multiradar wireless system was designed to track respiratory movements in real time. Through the two radar devices integrated on the linear accelerator, the movements of the chest and abdomen are monitored in real time (Gu et al., 2011; Gu and Li, 2015). However, the subjects need to spend much time and energy on training the breathing modes, which are required for the breathing exercise. One study printed markers as 11 circles on Lycra T-shirt (Ernst and Saß, 2015). Experiments demonstrated the advantages of depth camera over traditional optical acquisition equipment in measuring multiple moving targets, along with defects of image distortion and noise increase. Studies used RGB-D camera to collect continuous depth images of chest and abdomen regions of patients, with principal component analysis (PCA) used to create a respiratory motion model that displayed as tidal volume change (Wijenayake and Park, 2017). A method was proposed to estimate tidal volume changes by using depth camera to reconstruct three-dimensional isochronal surface of chest (Transue et al., 2016). The whole isosurface was extracted to characterize the deformation and volume changes of the patient's chest. Due to the inherent inconsistencies in the depth values provided by the depth camera, there are still problems such as depth measurement errors that need to be resolved.

The study of chest and abdomen surface measurement shows the advantages of the whole skin surface for the complete expression of motion information. Thus, to better model the motion of the whole thoracoabdominal surface and correlate, the surface information with the tumor motion is crucial. To obtain the respiratory motion characteristics of chest and abdomen surface, proper and accurate feature extraction is necessary. To the best knowledge of the authors, there are no available methods to obtain the feature extraction for the respiratory motion model comprised of voxels. However, our proposed method is inspired by previous research on feature extraction specially focusing on the classification and recognition in image processing. Studies used a convolutional network to train simple linear iterative clustering (SLIC) superpixels and obtained the feature embedding space of each superpixel, from which the superpixel feature vector was extracted (Liu et al., 2018). Some researchers used multifactor distribution as soft label and extracted supplementary information through visual input. Convolution neural network was used for visual feature learning, and the painting styles were classified through label distribution learning (Yang et al., 2018). In the research of data dimensionality reduction, locally linear embedding (LLE) and Gustafson–Kessel algorithms were used for dimension reduction in gray and RGB color images (Wang et al., 2018). A novel method introduced an end-to-end trainable and deep Siamese-like network named PersEmoN that consists of two convolutional network branches. Both networks share their bottom feature extraction module and are optimized within a multitask learning framework (Zhang et al., 2019). Experiments showed the effectiveness of PersEmoN on two apparent personality and emotion datasets.

**Figure 1:**
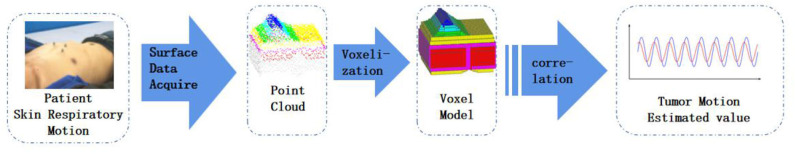
Flowchart of the respiration tracking system.

In this paper, based on our previous work on dynamic voxel modeling of thoracic–abdominal surface (Hou et al., 2019), a characterization method of respiratory motion was proposed, as shown in Figure 1. A visual information acquisition system with two depth cameras was established to collect the point cloud data of respiration movement. Afterwards, we constructed the point cloud surface into a watertight model using boundary interpolation. The watertight model was built into voxel model in the final step. Specifically, the established surface models based on time series are unprecedentedly to be transformed into voxel models with more three-dimensional structural information. Respiration features are extracted from voxel models by dimensionality reduction and used as a description of the whole thoracoabdominal torso. A correlation model between respiratory features and tumor motion was established. Finally, experimental results with model accuracy and correlation factor were studied to validate the proposed approach.

Compared with the solutions using limited number of infrared markers placed on the abdomen, the proposed method obtained more rich information about the patient's body surface by building 3D voxel models. A dimensionality reduction method based on 3D voxel model could extract more robust respiratory features from the established body surface model. It could also overcome the limitation of information loss problem that existed in current respiration tracking methods.

## Materials and Methods

The main framework of constructing correlation model is (1) establish dynamic thoracoabdominal surface voxel model, (2) reduce the dimension of the voxel model and extract the low-dimensional representation vector of the voxel model, and (3) establish the correlation model between the representation vector and the tumor motion state.

### Dynamic Thoracoabdominal Surface Voxel Modeling

Three-dimensional modeling of dynamic human thoracoabdominal skin surface during respiration mainly includes point cloud collecting of dynamic skin surface using multiple cameras of Kinect V2, model establishment of thoracoabdominal skin surface, and surface reconstruction into voxel model.

#### Point Cloud Acquisition System

With modeling integrity considered, we adopted time-series-based strategy when building the model with data from multiple cameras. Simultaneous frames of different cameras were fused into one frame, and then, the fused frames were arranged in time sequences. Due to the overlap in the fusion of two frames of time asynchrony, it is necessary to consider the time synchronization problem of multicamera sampling.

Since the exposure and trigger times of multiple cameras cannot be completely unified, we considered sampling with an approximate synchronization strategy. Two computers control two cameras in a one-to-one manner.

As images from multiple cameras have their own coordinates, an algorithm based on 2D image calibration was developed to unify the separate coordinates. In the algorithm, a calibration plate coordinate was built first by identification of the corner points, and then, the camera coordinate could be converted to the universal coordinates. Transformation from the camera coordinates system at any point *x*_*s*_ to the calibration plate coordinate system as *x*_*m*_ was as follows:

(1) $$\begin{array}{r} x_{m}=R_{s}\left(x_{s}-t_{s}\right) \end{array}$$

**Figure 2:**
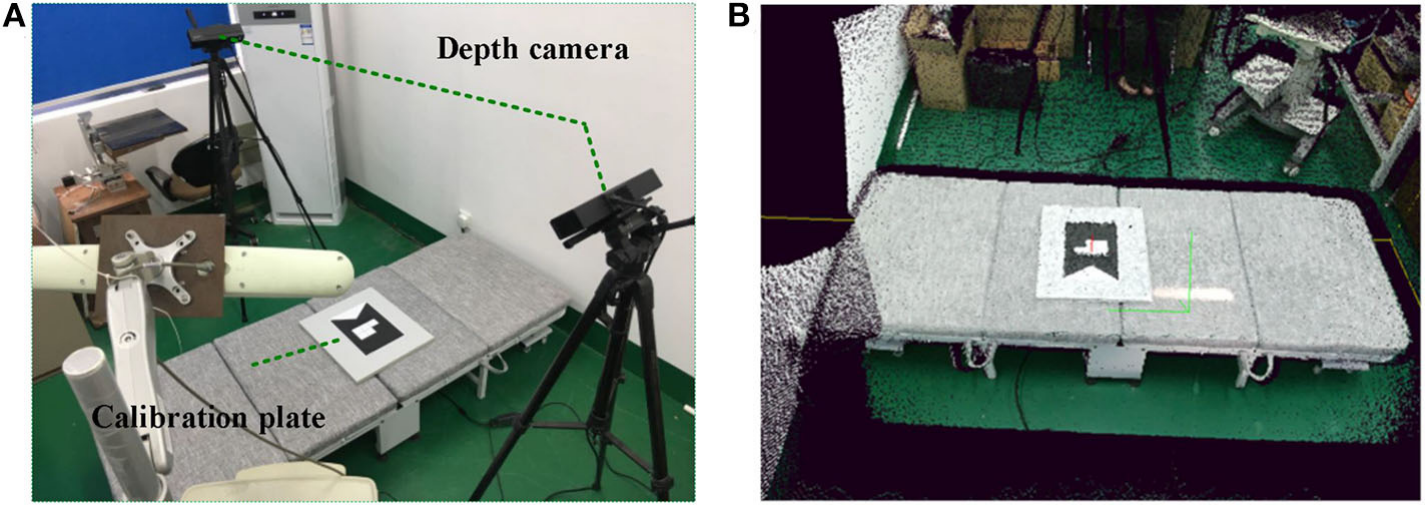
Calibration process for two Kinect V2 cameras. **(A)** Multicamera system. **(B)** Point cloud after calibration.

Among them, *t*_*s*_ is the center of the calibration plate in the camera coordinate system, and *R*_*s*_ is the rotation matrix. The position of the calibration plate in the world coordinate system is known, so any point can be converted from the coordinate system *x*_*m*_ to the world coordinate system *x*_*c*_:

(2) $$\begin{array}{r} x_{c}=R_{m}x_{m}+t_{m} \end{array}$$

where *R*_*m*_ and *t*_*m*_ are the rotation and translation of the calibration plate in the world coordinate system. Figure 2A shows the calibration setup consisting of two depth cameras and one calibration plate, and Figure 2B shows the point cloud data obtained through the above calibration procedures.

#### Modeling of Thoracoabdominal Surface With Respiratory Movement

Although the point clouds of multiple cameras have been unified into the same coordinate system, the raw data of point cloud has noises and outliers brought by cameras themselves and infrared interference between each other camera. To pre-process the raw data, we used bilateral filtering (Tomasi and Manduchi, 1998) in denoise and statistical filtering (Moore, 1978) to eliminate outliers.

Due to certain calibration errors, the registration of the adjusted multiple point clouds still has overlap, which requires precise registration. We used a classical point cloud registration algorithm iterative closest point (ICP) to register multiple point clouds. The basic principle of ICP algorithm is to find the nearest point (*p*_*i*_, *q*_*i*_) in the target point cloud *P* and the source point cloud *Q* according to certain constraint conditions and then calculate the optimal matching parameter *R* and *t* to minimize the error function *E*(*R, t*). The error function is:

(3) $$\begin{array}{r} E\left(R,t\right)=\frac{1}{n}\overset{n}{\underset{i=1}{\sum }}||q_{i}-\left(Rp_{i}+t\right)|{|}^{2} \end{array}$$

where *n* is the number of nearest point pairs, *p*_*i*_ is one point in the target point cloud *P*, *q*_*i*_ is the nearest point corresponding to *p*_*i*_ in the source point cloud *Q*, *R* is the rotation matrix, and *t* is the translation vector. Figure 2 shows the two-frame point cloud fusion.

In order to obtain the required model of thoracoabdominal surface region and reduce the computation of point cloud processing, the existing model needs to be segmented. Due to the fixed placement of multiple cameras and the fixed scene, this paper adopted a fast and convenient segmentation algorithm with distance and color thresholds. The chest and abdomen area of the lying subject and the position of the treatment bed are constrained, and the auxiliary limit of the color threshold is applied to divide the expected chest and abdomen surface area. The segmented surface is uneven and has burrs. In order to make the thoracoabdominal surface model smooth, point clouds need to be smoothed. In this paper, moving least squares method (Breitkopf et al., 2005) was used to smooth point clouds.

#### Watertight Thoracoabdominal Model Establishment and Voxelization

To present more available information for respiration movement feature extraction, the surface model was transformed into a watertight model first. The procedure mainly contains boundary estimation and boundary insertion. The purpose of transforming the point cloud into watertight model is to facilitate the follow-up research to analyze the three-dimensional structure of the chest and abdomen surface based on time series and to avoid the structural problems such as unsmooth boundary and voids on the surface caused by the unclosed model.

**Figure 3:**
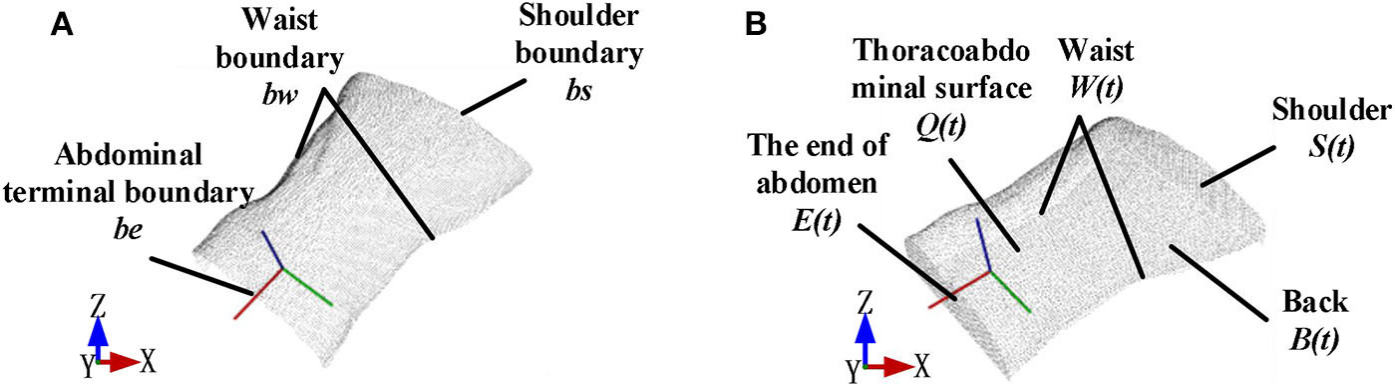
Point cloud interpolation to form a watertight model. **(A)** Boundary division before point cloud interpolation. **(B)** Watertight model after point cloud interpolation.

For boundary estimation, angles between searching points *p* and its adjacent points *p*_1_,…,*p*_*k*_ were adopted as criteria to detect the boundary. Threshold of the angle value was set to classify the boundary after angles of point *p*, and its neighbor points were calculated. Classified boundary points combined a set named as *Q*_*b*_. Figure 3A shows the boundary point sets *Q*_*b*_ were divided into *bw, bs*, *be* ∈ *Q*_*b*_, and Figure 3B shows the watertight thoracoabdominal model after point cloud interpolation.

**Figure 4:**
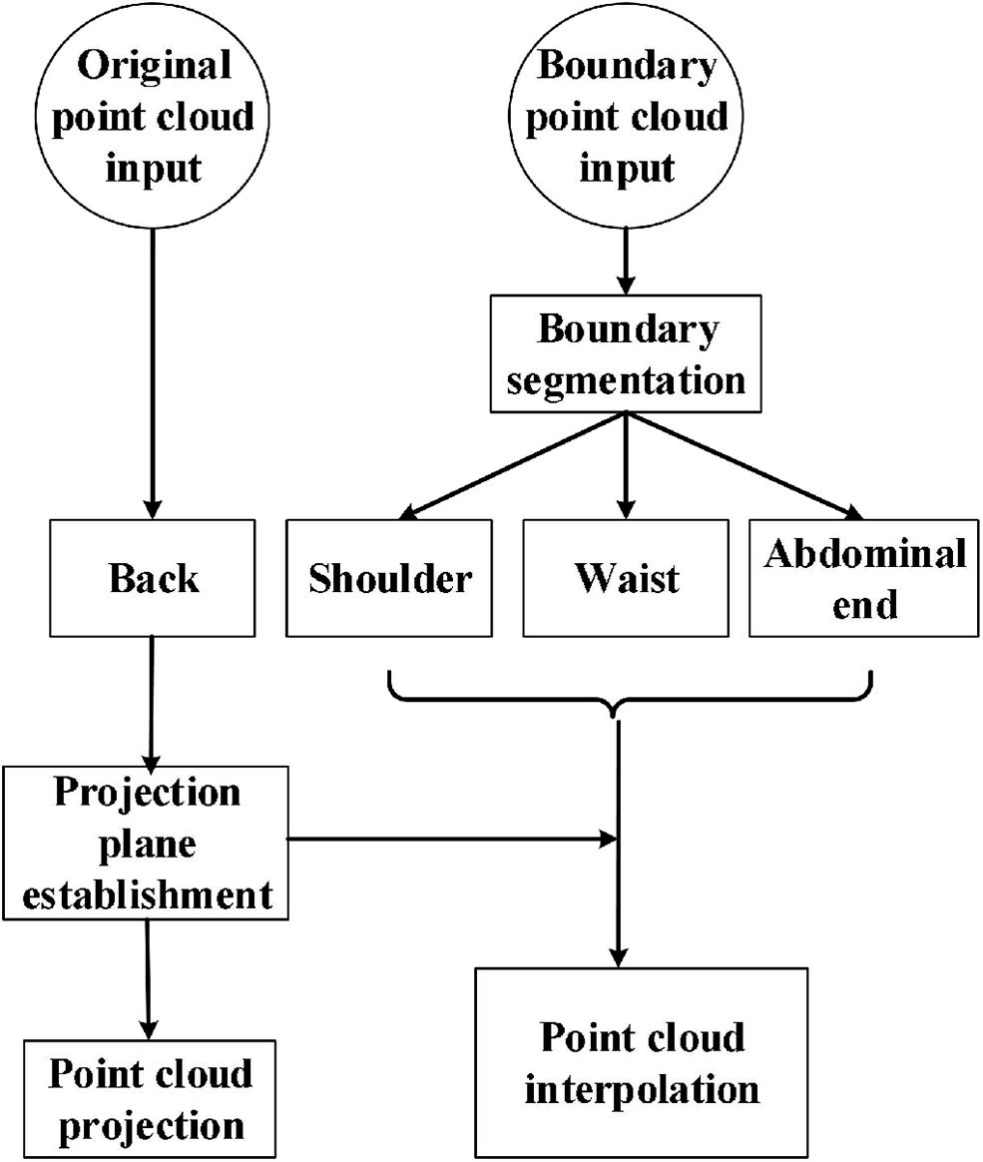
Flowchart of watertight model construction.

Figure 4 shows the workflow of the watertight model construction. First, the original point cloud input was used to generate the back part of the model. During the process, the projection plane was established as a *xy* projection plane *PL* that is 5 cm lower than the xy plane bounded by *bw* boundary. The back part of the point cloud set *B*(*t*) was built by projecting the corresponding points *q*_*i*_ ∈ *Q* in the original point cloud *Q* to the plane *PL*. To make the model watertight, boundary insertion was executed by using a method based on projection interpolation. As has been described above in Figure 3A, the boundary point cloud input was divided into the shoulder, waist, and abdominal end boundaries by boundary segmentation. After that, the shoulder part *S*(*t*) and the end of the abdomen *E*(*t*) was obtained by interpolating point cloud equally along the z negative axis to the plane *PL*, respectively; the waist part *W*(*t*) was obtained by interpolating point cloud equally along the waist curvature to the plane *PL*.

**Figure 5:**
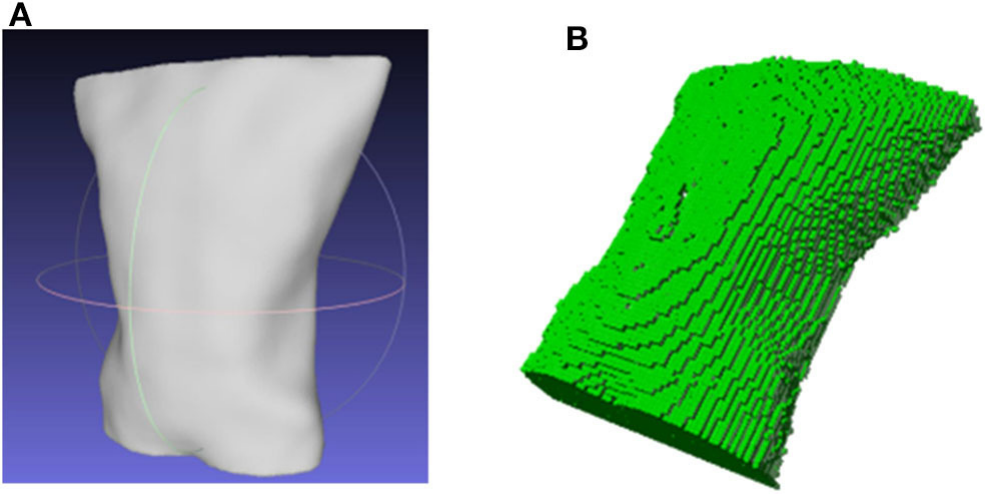
3D model of thoracoabdominal region. **(A)** Surface reconstruction. **(B)** Voxel model.

The watertight model composed of point cloud expressed the 3D information of the measured surface by independent points. To illustrate the space relationship of the entire surface, meshing was made to the point cloud model. Here, we used the classical Poisson reconstruction method (Kazhdan et al., 2006), in which normal vectors of the point clouds were calculated to display the curvature changes of the surface model. A smooth surface was built by estimation through indicator function's implicit fitting. Thus, the watertight thoracoabdominal point cloud model can be reconstructed into a model with smooth surface, as is shown in Figure 5A.

To study the volume changes of thoracoabdominal trunk movement, voxelization of the surface model is needed to convert the geometric representation of an object to the nearest voxel representation, which not only contains the surface information of the model but also describes the internal properties of the model. We used Octomap library (Hornung et al., 2013) to transform the point cloud into a voxel model. The voxelization of the model is shown in Figure 5B.

### Feature Vector of Dynamic Voxel Model

Due to the huge dimension of voxel model (arranging each voxel unit in columns up to several 100,000 dimensions), it is unrealistic to directly use these data to correlate with tumor motion because of a lot of noise and redundant information in huge data. In order to more effectively correlate with tumor motion and fully reflect surface motion characteristics, we first extract its physical significance features, then perform data analysis on the voxel model to extract respiratory features.

#### Physical Characteristics

The movement of breathing is accompanied by the heaving of the chest and abdomen. Intuitively speaking, the expansion and contraction of the chest and abdomen must cause the changes of the entire volume V and surface area S of thoracoabdominal trunk.

The entire voxel model's voxel number variation with time series reflects the volume change of the thoracoabdominal trunk. To study the respiration characteristic displayed by volume changes, the voxel units of voxel model of each frame were traversed, and then, the volume of voxel units was accumulated; therefore, the volume change is reflected in the time series. The area change of voxel model is reflected in the area of voxel's outer layer in the similar way.

#### Intrinsic Data Characteristics

Intrinsic data characteristics refer to the information that reveals the motional information of the body surface obtained by the voxel model. It is obtained by reducing the dimensionality of the voxel model. It is necessary for the construction of the respiration tracking model.

Just as point cloud generation came with time stamps, the state of voxel model varied over time series. Each frame of the voxel model stood for a respiratory state, which was expressed by probability of voxel occupancy. The overview of the proposed method was presented in Algorithm 1.

**Algorithm 1:** Construct Voxel-Bounding Box

**Input** : *M*(*t*) − *n*-frame voxel model
**Output** : *B*^′^(*t*) − *n*-frame modified-bounding box
1: **for** *M*_*i*_ ∈ *M*(*t*) **do**
2: Set the probability of each voxel in *M*_*i*_ to 1
3: Calculate the length, width, and height of the bounding box of *M*_*i*_ as: *L*_*Mi*_ ∈ *L*, *W*_*Mi*_ ∈ *W*, *H*_*Mi*_ ∈ *H*
4: **end for**
5: Search for the maximum value of length, width and height respectively as *L*_max_, *W*_max_, *H*_max_, to form a template-bounding box *B*
6: Divide *B* into space units that decided by the resolution of each voxel
7: **for** *M*_*i*_ ∈ *M*(*t*) **do**
8: Compare the template-bounding box *B* and *M*_*i*_ to obtain their intersections. In template-bounding box *B*, set the probability of the intersection voxels to 1 and set those of the rest voxels in to 0, to obtain the modified bounding box *B*_*i*_^′^
9: **end for**

**Figure 6:**
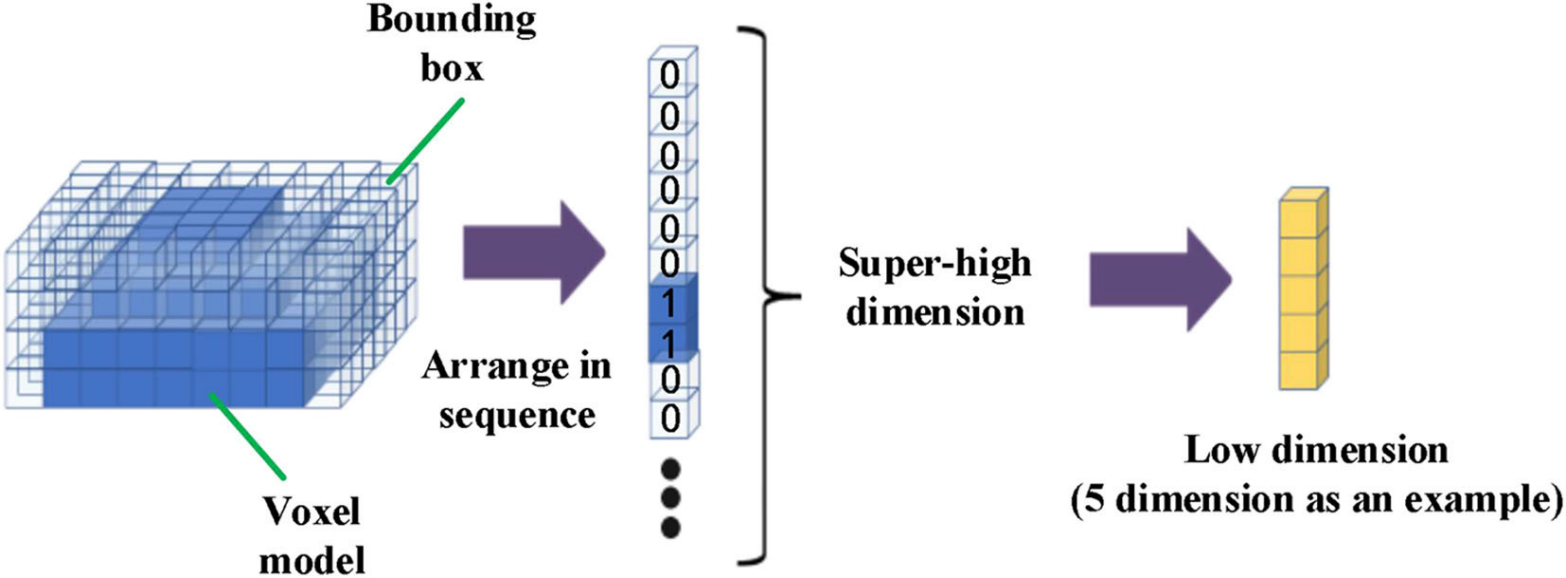
Flowchart of voxel model dimensionality reduction.

Depending on the voxels' occupation and idleness, the state of voxels in the template-bounding box were marked with probability 1, 0, respectively. Thus, the voxel model state varied by time was expressed by the marker value of each voxel in the template-bounding box, as shown in the left diagram in Figure 6. Since the bounding box contained a large number of voxels, which could be illustrated as columns of a superhigh-dimensional vector, it would cost huge calculation if the original vectors were used to build the correlation model. Therefore, the vector Υ with superhigh dimension was transformed into a low-dimensional vector ψ that remained the characteristics of the voxel model changes. To accomplish dimension reduction, an algorithm based on LLE (Roweis, 2000) is shown in Algorithm 2.

**Algorithm 2:** Respiration Representation Based on LLE

**Input** : *B*^′^(*t*) − *n*-frame modified-bounding box
**Output** : ψ − Low-dimensional vector
1: **for** *B_i_*^′^ ∈ *B*^′^(*t*) **do**
2: Define a column vector *x*_*i*_ by the probability arrangement in a certain spatial order of each voxel in bounding box *B_i_*^′^, *x*_*i*_ ∈ *X*
3: **end for**
4: Obtain a sample space *X* = {*x*_*1*_, *x*_*2*_, …, *x*_*n*_} as input for dimensionality reduction
5: **for** *x*_*i*_ ∈ *X* **do**
6: Search for the *K* nearest neighbors of each sample *x*_*i*_
7: Construct the loss function of the mean square error of *x*_*i*_ and the linear representation of its *K* nearest neighbors and normalize the weight coefficients
8: Solve the optimal solution of the loss function as the weight coefficient vector *W*_*i*_ of each sample *x*_*i*_
9: **end for**
10: Weight coefficient matrix *W* is composed of obtained *W*_*i*_
11: Calculate *M* = (*I* − *W*)(*I* − *W*)^*T*^ and solve the eigenvalues and eigenvectors of *M*
12: Choose the optimal dimension *d* considering the contribution rate of eigenvalue and obtain the sample matrix with lower dimension as ψ = {ψ_2_, ψ_3_, …, ψ_*d*+1_}

Through combining physical variables *V*, *S* and essential parameters ψ = [ψ_1_, ψ_2_, .., ψ_*m*_](*m* ≤ *d* + 1) after data analysis, characteristic variables Γ that can express time-varied states of the voxel model can be obtained as:

(4) $$\begin{array}{r} \Gamma ={\left[V,S\ldots \psi_{1},\psi_{2},..,\psi_{m}\right]}^{T} \end{array}$$

### Correlation Between Characterization Vector and Tumor Motion

After feature extraction of voxel model in previous section, we obtained the representation vector of the whole thoracoabdominal surface motion. The establishment of respiratory correlation model is a key part of tumor tracking. The correlation function is to take the breathing surrogate signal (the characteristic signal of skin surface motion that is highly correlated with tumor motion in the body) as the input data and realize the motion estimation of the internal tumor by correlating the surrogate with the movement of the internal tumor. Therefore, we used the extracted representation vector as external surface motion surrogate to establish a correlation model with internal tumor motion.

Tumor movement is complex since it is caused by various factors such as respiration, heartbeat, and abdominal pressure. During inhalation, the volume of air inhaled by the lungs becomes larger because of the thoracoabdominal cavity and the diaphragm deform. During the exhalation process, the volume of gas remaining in the lungs becomes smaller; at the same time, the thoracoabdominal cavity and diaphragm are restored to their original state. During normal breathing, at the same transpulmonary pressure, the expiratory volume is greater than the inspiratory volume. The so-called hysteresis phenomenon (the phase lag between body surface characteristic respiratory motion and tumor respiratory motion) is attributed to the complex respiratory pressure–volume relationship of the lungs and chest and abdomen.

Therefore, exhalation and inhalation are two irreversible motion states. Before establishing the correlation model, we need to divide the entire breathing process into exhalation part and inhalation part and model these two processes, respectively. This paper divides the respiratory phase according to the amplitude of the external motion and uses the peak and valley values of the motion as the dividing points of exhalation and inspiration.

Due to the non-linear relationship between internal (the motion information of tumor *in vivo*) and external motion (the motion information of body surface), we adopt a polynomial model (Peressutti et al., 2012). That is, the trajectory of the tumor *in vivo* is approximated as a linear combination of multiple power terms of the external signal. In this paper, different polynomial functions are used to model the breathing movement during the exhalation and inhalation phases:

(5) $$\begin{array}{r} X_{T_{i}}=\{\begin{array}{c} \overset{N}{\underset{j=0}{\sum }}A_{j}^{+}r_{i}^{j},r\geq k_{i}\\ \overset{N}{\underset{j=0}{\sum }}A_{j}^{-}r_{i}^{j},r\leq k_{i} \end{array} \end{array}$$

in which *A*_*j*_ is a polynomial coefficient, *N* is the highest power, and *k*_*i*_ is the dividing points of each expiratory and inspiratory process. The highest power of the polynomial model is preferably 2 or 3. Higher powers are prone to overfitting and reduce the generalization ability of the model.

## Experiments and Results

Experiments have been carried out to verify the feasibility of the respiratory motion characterization method based on thoracoabdominal surface modeling. Our experiments mainly focused on two aspects as validity of thoracoabdominal surface described by point cloud and validity of feature extraction on surface–tumor correlation. *Position Comparison of Multiple Markers* and *Comparison of Marker Position Obtained by Point Cloud and NDI Method* validated that whether the representation method of skin surface modeling has the same effect as the traditional representation method of finite markers; *Optimization of Dimension Reduction* and *Correlation Coefficient of Dimension-Reduced Feature Vector and NDI Markers With Tumor Motion* validated whether this method has more comprehensive characterization capability than the traditional finite marker method; in *Correlation Model of Dimension-Reduced Feature and Tumor*, correlation model between dimension-reduced features of thoracoabdominal voxel data and tumor motion was built and compared with the traditional method.

**Figure 7:**
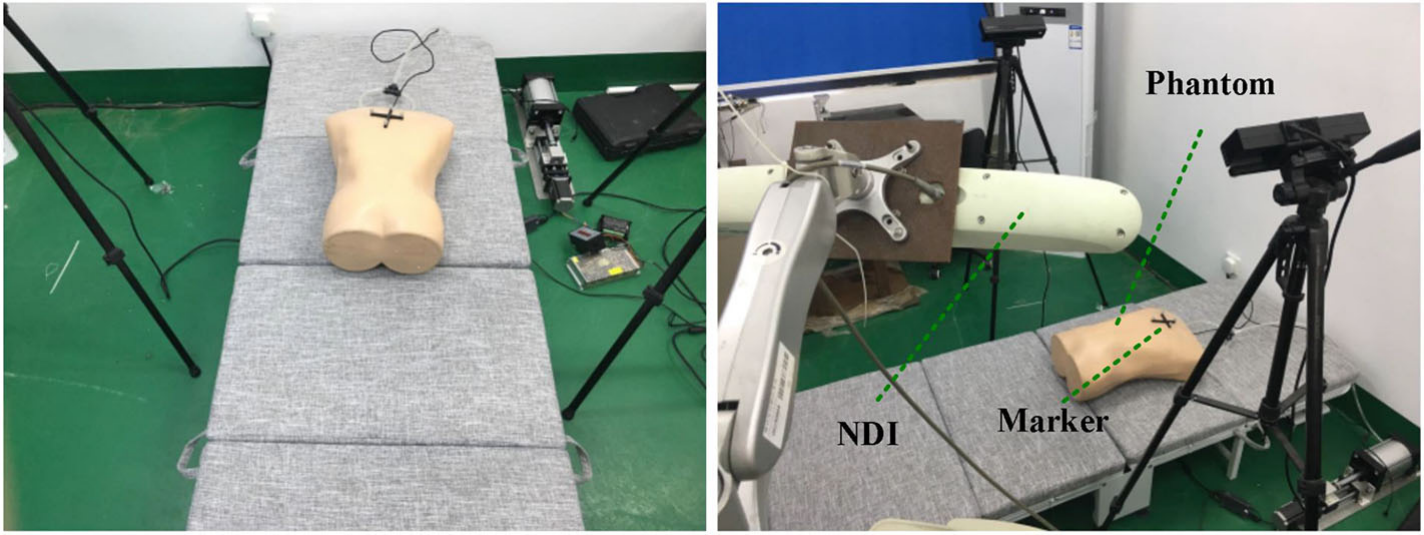
Scene of NDI marker experimental data acquisition.

We used two Kinect V2 (Microsoft Co.) depth cameras placed at both sides of the experimental bed to collect data. Because the view of a single camera is limited, blind areas cannot be observed on the abdomen exit and will prevent a successful 3D modeling of thoracic–abdominal surface. However, using two Kinect V2 cameras has a complete view of the patient's body and is conducive to building the whole thoracic–abdominal model. The experimental subject is a phantom developed in our lab (Hou et al., 2018) for simulating respiratory movement of thoracoabdominal cavity. The experimental scene is shown in Figure 7. In order to compare the skin surface characterization method with traditional markers, we used NDI Polaris Spectra (Northern Digital Inc.), an optical tracking system (accuracy, 0.25 mm RMS) to record the skin markers' movement. Before experiments, we need to calibrate the coordinate systems of NDI Polaris Spectra and Kinect V2 depth cameras. To unify the two coordinate systems of point cloud and NDI system, spherical center of NDI optical markers in point cloud data was fitted to match with the marker's coordinates obtained by NDI Polaris Spectra.

### Position Comparison of Multiple Markers

**Figure 8:**
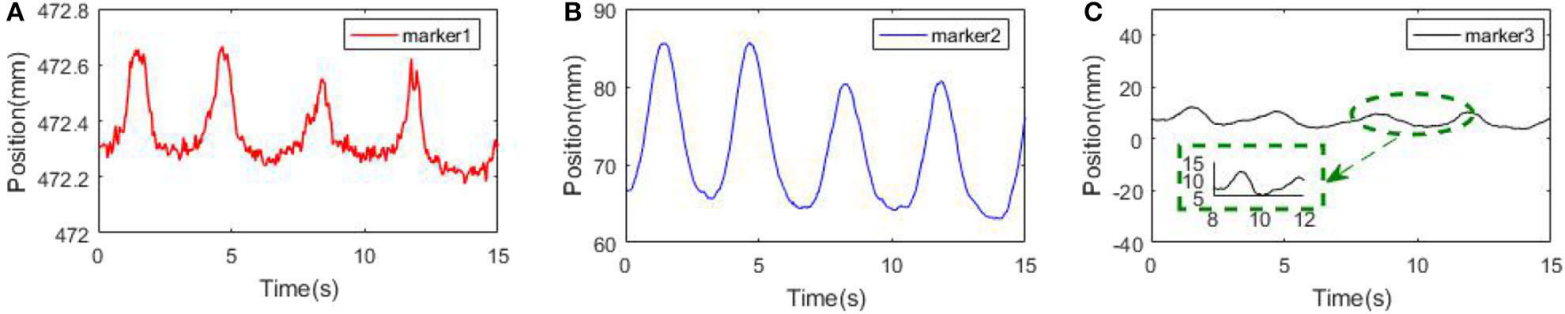
Principal component motion of surface markers. **(A)** Marker 1. **(B)** Marker 2. **(C)** Marker 3.

In order to display the differences among multiple markers in characterizing the respiratory movement, the position of the markers on the chest and abdomen surface is changed, and the NDI sensor is utilized to track the motion change. Figure 8 shows the principal movement of the markers analyzed by PCA at different positions. Although the frequency and phase of the three markers are the same, the maximum motion range of marker 1 is about 5 mm, the maximum motion range of marker 2 is about 2 cm, and the maximum motion range of marker 3 is about 1 cm. The markers at different positions reflect different movements. It can be inferred that limited skin surface markers are not complete in motion representation of the whole thoracoabdominal surface.

### Comparison of Marker Position Obtained by Point Cloud and NDI Method

To clarify whether our characterization method based on thoracoabdominal surface modeling contains the information provided by traditional finite markers, we made comparisons of the same marker position obtained by Kinect V2 and NDI optical tracking system. Before the comparison experiments, we need to unify the point cloud data into the NDI coordinates system. Therefore, calibration of NDI coordinates system and Kinect V2 coordinate system was carried out as follows.

Suppose the transformation equation of the two coordinate systems is *A* = *RB* + *T*, where *A* is the position of one point in coordinates of NDI, *R* is the rotation matrix, *B* is the coordinates of the same point in the point cloud coordinate system, and *T* is the translation matrix. To solve *R* and *T*, three markers were placed on the chest of the phantom for five times, each time with different positions, respectively. NDI optical tracking system and Kinect V2 recorded data simultaneously at the five experiments. After collecting the data, NDI data were used to calculate the corresponding transformation matrix *R, T*. Then, the point cloud obtained by Kinect V2 system was compared with the position of the marker under the NDI sensor.

**Figure 9:**
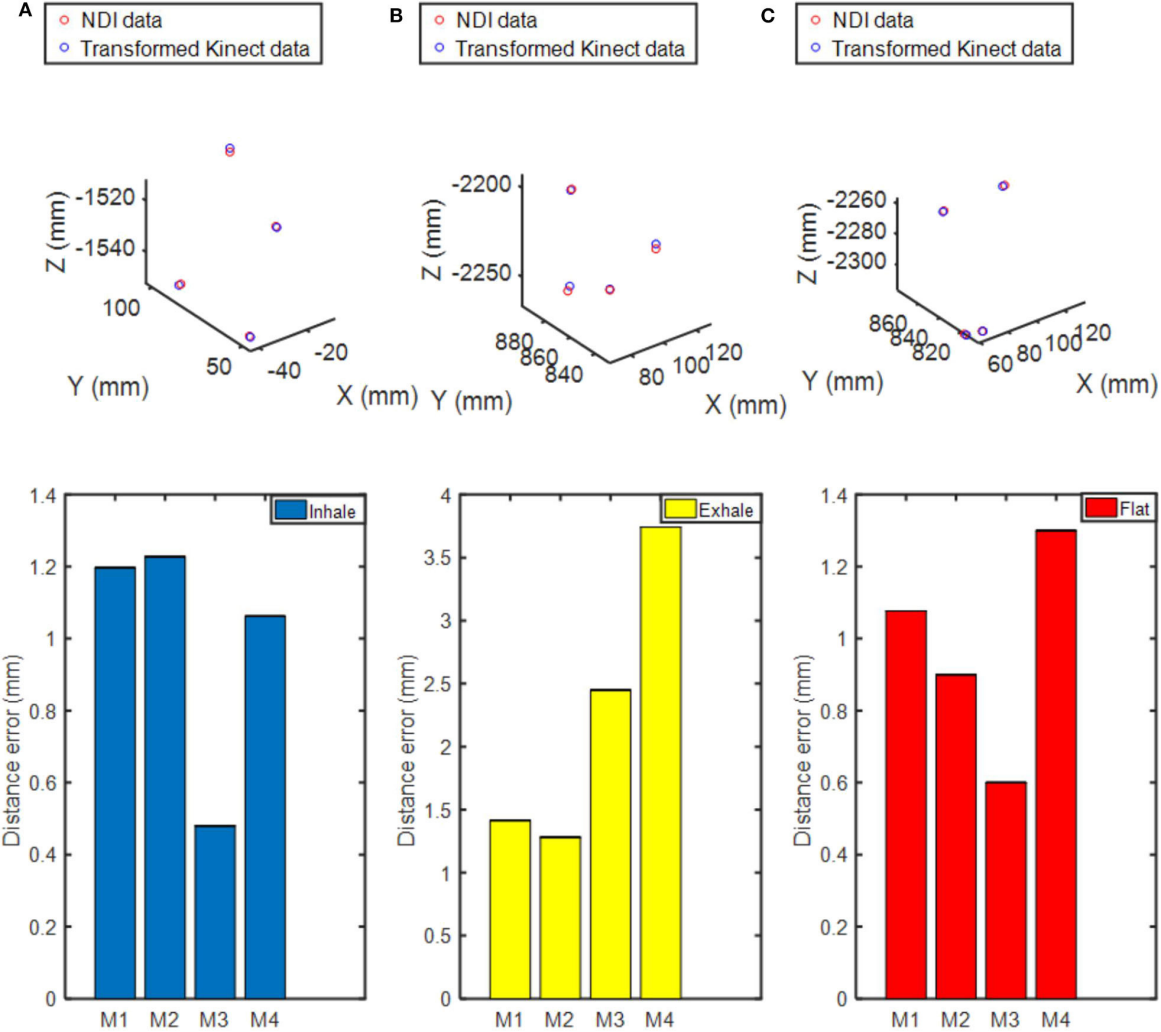
Comparison of the spatial position of the markers in the depth camera coordinate system and the NDI system coordinate system. **(A)** Inhalation. **(B)** Exhalation. **(C)** Transition between inhalation and exhalation. The uppermost figure describes the spatial position of the NDI markers and the transformed ones from point cloud data in the NDI system coordinate. The figure below shows the distance error between the transformed position and the actual NDI position.

Center positions of four NDI markers' by fitting of point cloud obtained by Kinect V2 system were transformed and compared with the position of NDI markers under NDI sensor at three breathing fractions. As shown in Figure 9, there are three respiratory states: inhalation, exhalation, and transition between inhalation and exhalation. The uppermost figure represents the position relationship between the two coordinates in the three-dimensional space after conversion. By comparing the Euclidean distance between the two coordinates, it can be seen from the figure that the maximum distance error between the two coordinates is within 4 mm. The coordinates of the two can basically correspond to each other through conversion. It can be concluded that the characterization method based on thoracoabdominal surface modeling has the same effect as the traditional characterization method of finite markers.

### Optimization of Dimension Reduction

**Figure 10:**
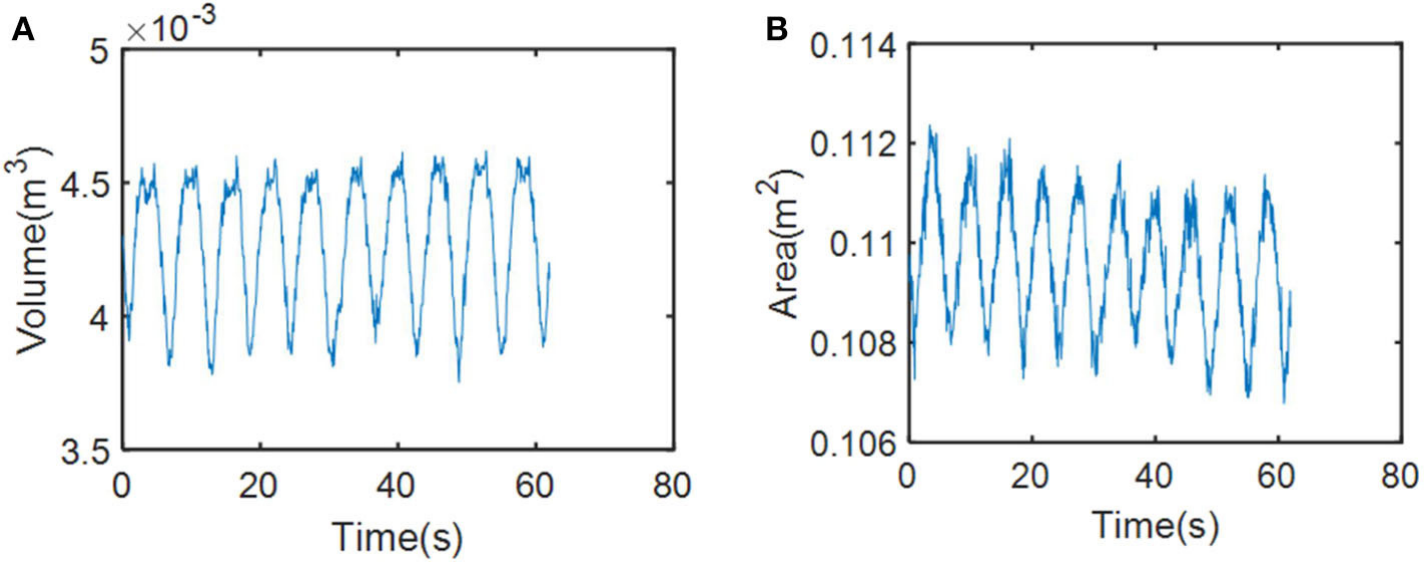
Changes in respiratory motion of physical characteristics. **(A)** Changes in respiratory motion of volume feature. **(B)** Changes in respiratory motion of area feature.

**Figure 11:**
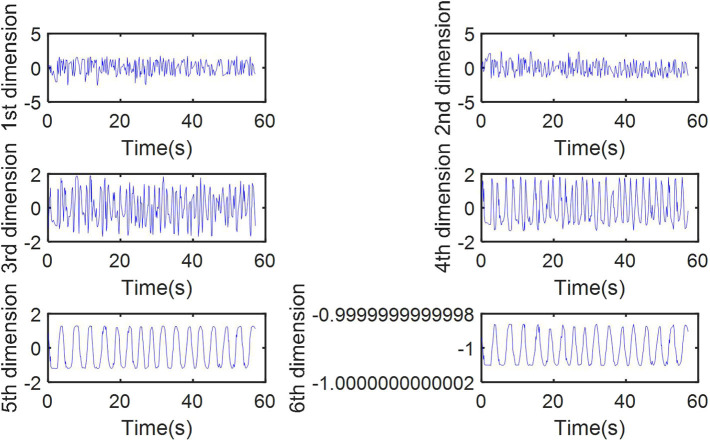
Respiratory motion features of thoracoabdominal model in six dimensions after reduction.

Unlike the traditional method of representing several isolated points, the advantage of our method lies in characterizing the entire surface feature. We collected respiratory motion data from our phantom for 1 min and obtained voxel models based on time series through the modeling method described in *Dynamic Thoracoabdominal Surface Voxel Modeling*. Figure 10 shows the volume and area features, respectively, extracted by using the method mentioned above. We can see that volume and area characteristics display periodicity and rhythmicity and conform to the law of respiratory movement. Then, we used dimension reduction analysis of voxel model proposed in *Feature Vector of Dynamic Voxel Model*. The bounding box contains 172,530 voxel units with a resolution of 5 mm and constitutes a 172,530-dimensional column vector, which can be reduced to any dimension. For example, we reduced the high-dimensional data to a characteristic vector with six dimensions. The respiratory characteristics in six dimensions are shown in Figure 11.

Whether the results of dimensionality reduction can accurately reflect the respiratory movement of the surface needs to be validated. In addition, optimal number of the characteristic vector after dimension reduction needs to be ascertained. To solve these problems, Pearson correlation method (Person, 1895) was used for correlation analysis in this paper. The formula is as follows:

(6) $$\begin{array}{r} \rho_{X,Y}=\frac{cov\left(X,Y\right)}{\sigma_{X}\sigma_{Y}} \end{array}$$

where the Pearson correlation coefficient ρ_*X, Y*_ of two continuous variables (*X, Y*) is equal to the covariance *cov*(*X, Y*) between them divided by the product of their respective standard deviations (σ_*X*_, σ_*Y*_). The value of the coefficient is always between −1.0 and 1.0. Variables close to 0 are regarded as uncorrelated, while variables close to 1 or −1 are regarded as strongly correlated.

Three sets of phantom data were, respectively, used to build voxel models and were reduced into characteristic vectors with 10 dimensions first. Then, correlation analysis between each dimension and tumor movement *in vivo* is carried out using Equation (6). As shown in Table 1, there is only one dimension with a correlation coefficient >0.5 with tumor motion *in vivo*. The correlation coefficient between this only dimension and tumor *in vivo* reached more than 0.9. Therefore, we can conclude that reducing the superhigh-dimensional voxel model to 1 dimension is the optimal operation.

**Table 1:** The correlation coefficient between each dimension and tumor motion.

	**Pearson correlation coefficient**									
**Phantom data**	**1st**	**2nd**	**3rd**	**4th**	**5th**	**6th**	**7th**	**8th**	**9th**	**10th**
P1	0.0427	0.0135	0.0912	0.0363	0.0119	0.0177	0.1188	0.0353	0.0141	0.9230
P2	0.0262	0.0160	0.0521	0.0023	0.0940	0.0355	0.1030	0.1926	0.0039	0.9050
P3	0.0872	0.0393	0.0311	0.0690	0.1734	0.1176	0.0280	0.0215	0.0476	0.9491

*The Pearson correlation coefficient between each dimension and tumor motion is calculated in the table*.

### Correlation Coefficient of Dimension-Reduced Feature Vector and NDI Markers With Tumor Motion

In order to verify the correlation between each variable and tumor movement, we also calculated the correlation coefficient between tumor motion, dimension-reduced data, and NDI markers, respectively, by using Pearson method as Equation (6). The results are in Table 2. The correlation coefficient between the dimension-reduced feature vector and tumor motion is higher than that of skin surface markers and tumor motion. To sum up, the movement of chest and abdomen surface can be featured by one-dimensional feature vector. Besides, fusion of multidimensional voxel model data represents movement of chest and abdomen surface more accurately than markers.

**Table 2:** The correlation coefficient between the dimensionality reduction variable and NDI marker.

	**Pearson correlation coefficient**	
**Phantom data**	**NDI marker to tumor**	**Dimension-reduced feature to tumor**
P1	0.8553	0.9141
P2	0.8369	0.9039
P3	0.8419	0.9476

*The Pearson correlation coefficient between each parameter and tumor motion is calculated in the table*.

### Correlation Model of Dimension-Reduced Feature and Tumor

As the correlation model has been introduced in the previous section, one-dimensional data were obtained by the dimension reduction method to establish the correlation model with tumor motion *in vivo*.

The obtained data (last for 1 min) is divide into two parts: one part was used to establish the correlation model, and the other one was used to verify the accuracy of the correlation model. That is, the correlation model was used to estimate the tumor motion *in vivo* using skin surface motion, while the feasibility and accuracy of the method were proved by comparing the error between the actual tumor motion and the estimated value.

**Figure 12:**
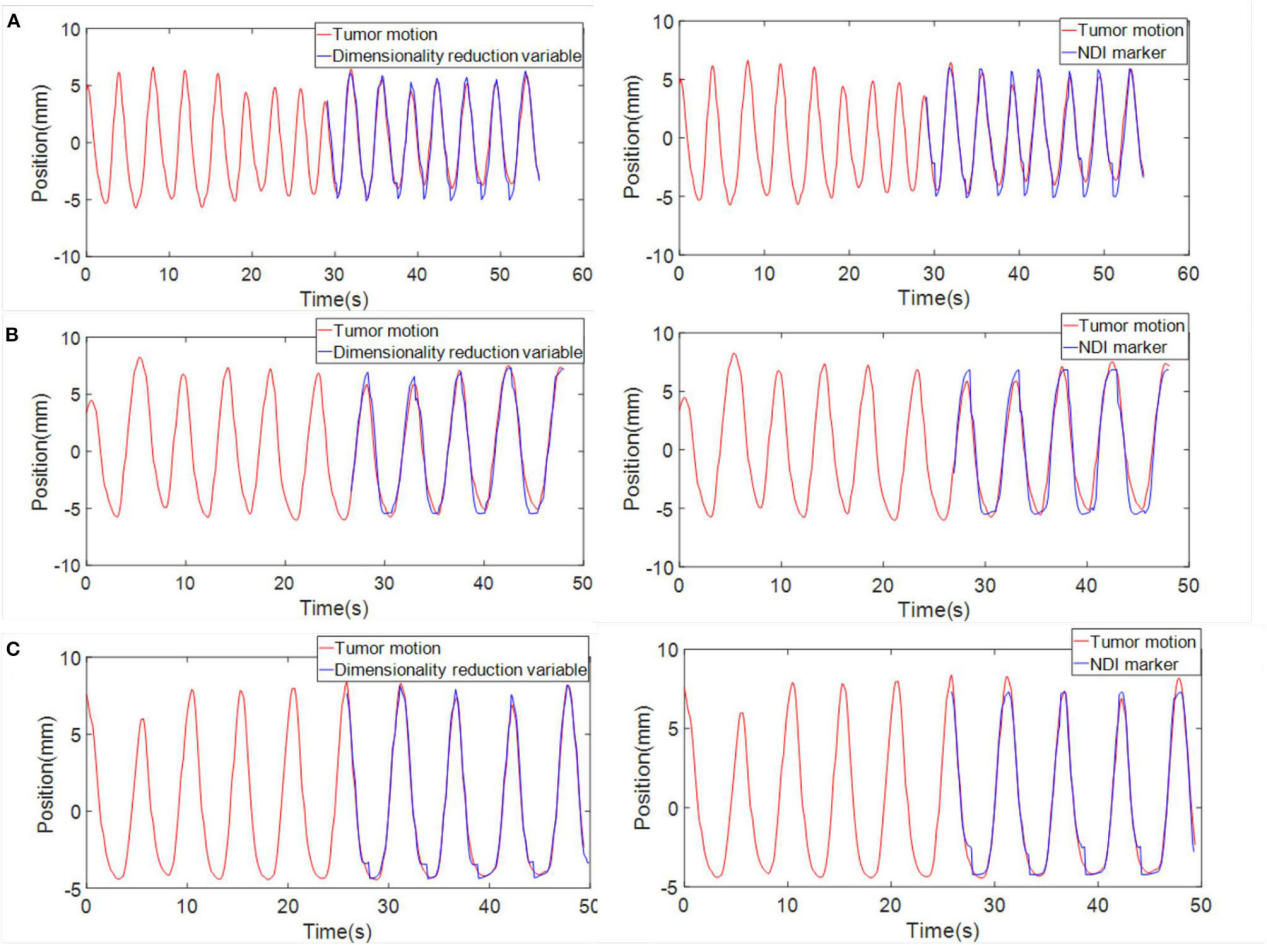
Comparison between the tumor motion estimation and their actual value (red, actual value; blue, estimated value). **(A)** Phantom data 1. **(B)** Phantom data 2. **(C)** Phantom data 3. Three sets of data are displayed. Curves in the left column show the estimation of tumor motion obtained through the correlation model built with dimensionality reduction results. Curves in the right column shows the estimation of tumor motion when NDI marker position was chosen as the input of the correlation model.

Then, we established the correlation model with tumor motion by using the dimensionality reduction results and NDI marker positions, respectively. After that, correlation models were used to estimate tumor motion. Three sets of phantom data with point cloud collection and NDI maker positions were used to test and compare the two correlation models. Figure 12 shows the comparison results. The mean squared error (MSE) between the estimated and actual values was also calculated and presented in Table 3. From Table 3, it can be seen that the accuracy of the correlation model obtained by dimension reduction results is much higher than the one of the correlation model obtained by NDI marker, which also indicates that the proposed approach in this work is feasible to extract respiratory features based on horacoabdominal surface modeling, and it is capable to establish an enhanced respiratory correlation model than skin surface marker method.

**Table 3:** The mean squared error (MSE) error between the estimated and actual values.

	**MSE error(mm)**	
**Phantom data**	**NDI Marker**	**Dimensionality reduction variable**
**1**	0.0136	0.0117
**2**	0.0082	0.0055
**3**	0.0051	0.0037

## Discussion and Conclusion

In this paper, a novel characterization method of human external respiratory motion for tumor correlation was proposed. Compared with previous methods of limited markers and multiple modality surrogates, our method accomplished better characterization by establishing a 3D voxel model containing rich structural information of thoracic and abdominal torso during the respiratory motion. Dimensions of the voxel model were reduced by a dimensionality reduction method. Through these procedures, features that can reflect changes in external respiratory motion were obtained. This paper builds the model of chest and abdomen torso's respiratory motion, from multicamera system construction, point cloud processing, and watertight model construction to model voxelization. A dimensionality reduction method was proposed to extract respiratory features from the established voxel model. The feasibility of the characterization method based on chest and abdomen surface modeling was verified and compared with the traditional finite surface marker representation method. The proposed method was proved to be more accurate than the traditional one with limited external markers. Furthermore, it breaks through the limitation of information loss in current respiration correlation methods, which describe the respiratory motion by using sparse sensing data. In future work, to form a sound solution of respiration tracking for radiosurgical robots, we will optimize the process of thoracoabdominal skin surface modeling, study the feature extraction of voxel model, and apply this method to the prediction of tumor motion under the clinical application of tumor tracking.

## Data Availability Statement

All datasets generated for this study are included in the article/supplementary material.

## Ethics Statement

Ethical review and approval was not required for the study on human participants in accordance with the local legislation and institutional requirements. The patients/participants provided their written informed consent to participate in this study. Written informed consent was obtained from the individual(s) for the publication of any potentially identifiable images or data included in this article.

## Author Contributions

SY contributed to the manuscript writing, data analysis, and experiment design. PH contributed to the manuscript writing and data analysis. RS conceived the presented idea and contributed to the algorithm design. MZ and JG contributed to the manuscript revision and system design. FZ, SK, and LS contributed to the support of experimental equipment. All authors discussed the results and contributed to the final manuscript.

## Conflict of Interest

The authors declare that the research was conducted in the absence of any commercial or financial relationships that could be construed as a potential conflict of interest.
